# Mouse decellularised liver scaffold improves human embryonic and induced pluripotent stem cells differentiation into hepatocyte-like cells

**DOI:** 10.1371/journal.pone.0189586

**Published:** 2017-12-20

**Authors:** Maëlle Lorvellec, Federico Scottoni, Claire Crowley, Rebeca Fiadeiro, Panagiotis Maghsoudlou, Alessandro Filippo Pellegata, Francesca Mazzacuva, Asllan Gjinovci, Anne-Marie Lyne, Justine Zulini, Daniel Little, Olukunbi Mosaku, Deirdre Kelly, Paolo De Coppi, Paul Gissen

**Affiliations:** 1 MRC Laboratory for Molecular Cell Biology, University College London, London, United Kingdom; 2 Department of Stem Cells and Regenerative Medicine, Institute of Child Health and Great Ormond Street Hospital, University College London, London, United Kingdom; 3 Centre for General Omics, Institute of Child Health and Great Ormond Street Hospital, University College London, London, United Kingdom; 4 Institut Curie, PSL Research University, Paris, France; 5 The Liver Unit, Birmingham Children’s Hospital, Birmingham, United Kingdom; Wake Forest Institute for Regenerative Medicine, UNITED STATES

## Abstract

Liver transplantation is the definitive treatment of liver failure but donor organ shortage limits its availability. Stem cells are highly expandable and have the potential to differentiate into any specialist cell. Use of patient-derived induced Pluripotent Stem Cells (hiPSCs) has the additional advantage for organ regeneration therapies by removing the need for immunosuppression. We compared hepatocyte differentiation of human embryonic stem cells (hESCs) and hiPSCs in a mouse decellularised liver scaffold (3D) with standard *in vitro* protocol (2D). Mouse livers were decellularised preserving micro-architecture, blood vessel network and extracellular matrix. hESCs and hiPSCs were primed towards the definitive endoderm. Cells were then seeded either in 3D or 2D cultures and the hepatocyte differentiation was continued. Both hESCs and hiPSCs differentiated more efficiently in 3D than in 2D, with higher and earlier expression of mature hepatocyte marker albumin, lipid and glycogen synthesis associated with a decrease in expression of fetal hepatocyte marker alpha-fetoprotein. Thus we conclude that stem cell hepatocyte differentiation in 3D culture promotes faster cell maturation. This finding suggests that optimised 3D protocols could allow generation of mature liver cells not achieved so far in standard 2D conditions and lead to improvement in cell models of liver disease and regenerative medicine applications.

## Introduction

Liver disease is the third most common cause of premature death in the UK. In 2012, in England and Wales, 600,000 people had some form of liver disease [1] whilst in 2013, it affected 30 million Americans [2]. Liver disease includes alcohol-related liver disease, viral hepatitis, non-alcoholic fatty liver disease, autoimmune and inherited liver disorders. In 2013, 29 million Europeans had a form of chronic liver condition [3]. Globally, liver cirrhosis was responsible for over one million deaths which correspond to approximately 2% of all deaths worldwide [4], in 2010; and 10,948 deaths were due to liver cirrhosis, in 2012, in the UK [1]. Liver transplantation is the primary treatment for severe liver failure; however, it is currently limited by organ shortage [5].

Over the last few decades, a number of alternative approaches to liver transplantation have been developed including bioartificial liver support systems (BAL) and primary hepatocytes transplantation. The use of BAL (extracorporeal bioreactors in which liver cells are cultured in a network of hollow fibres for blood plasma perfusion) is limited by the lack of a reliable, safe, metabolically active and easily expandable human cell source. Furthermore, BAL membranes do not provide an appropriate molecule exchange mechanism [6]. In a recent study, Shi et al used human fibroblasts directly reprogrammed into human hepatocytes in a BAL support system and showed promising results in metabolic detoxification and ammonia elimination. However, the cells used did not acquire many of the mature hepatocyte functions and the exchange between blood, plasma and cells remained limited [7]. On the other hand, hepatocyte transplantation has shown promise as a short-term treatment for specific metabolic liver disorders; however, as yet, this approach has not had a significant impact upon treatment of acute liver failure and chronic liver disease. The application of this treatment modality is limited by the lack of a sufficient source of viable primary hepatocytes and poor cell engraftment in situ [6, 8, 9].

A possible answer to the lack of an easily expandable human cell source is the use of patient-derived induced Pluripotent Stem cells (hiPSCs) [10]. These cells have the potential to differentiate into any cell type [11] and the added advantage of removing the need for immunosuppression. hiPSCs differentiation protocols utilised so far [12–14] have the limitation to allow cells to achieve only fetal-like hepatocyte phenotype [13, 15], whereby the cells lack any ability to perform many mature hepatocyte functions. Full maturation to an adult phenotype is however necessary to allow transplantation of these cells in an adult setting.

Normal cell physiology and function strongly depend on cell-cell and cell-extracellular matrix (ECM) interactions in the 3D environment. Several studies have demonstrated that primary human hepatocytes (PHH) grown in 3D systems [16–18] maintain the state of differentiation better as evidenced by improved function, enhanced cell polarization and canalicular formation. Gieseck et al showed that hiPSC-derived hepatocytes grown in the RAFT (3D cell clumps in 3D Real Architecture For 3D Tissues) system had a more mature phenotype than those grown in 2D with more prominent intercellular junctions [19, 20]. Besides the mechanical stimuli related to the 3D environment, naturally derived ECM has also the advantage of delivering tissue-specific signalling which have a role in the regulation of liver cell function [21], migration and proliferation [22]. In particular key components of ECM, such as laminin, may assist the differentiation of stem cells into hepatocytes [23]. As a consequence, fewer cytokines and growth factors may be required in the media for hepatocyte differentiation from human hepatic stem cells cultured in scaffolds after decellularisation than on cells grown on collagen type I -coated dishes [24]. This influence is so powerful that in fetal and adult hepatocytes, it promotes expression of adult genes in adult hepatocyte ECM or the expression of fetal genes in fetal hepatocyte ECM, respectively [25].

In this study, we tested mouse decellularised liver scaffolds as a platform for stem cells to hepatocyte differentiation in order to combine the advantages of a 3D environment with the benefits provided by the ECM components.

A mouse decellularised liver scaffold, which we have previously shown to preserve extracellular matrix components and fine hepatic microarchitecture, was used to seed previously endoderm stage primed hiPSCs and hESCs [26, 27] (3D cultures). Mouse decellularised liver scaffolds are easily available and can be repopulated with a relatively small amount of cells compared to human decellularised liver scaffolds. The differentiation stage of the stem cells cultured in 3D was compared to that of cells that underwent a conventional differentiation protocol on matrigel-coated dishes (2D cultures) by analysis of hepatocyte markers expression.

## Materials and methods

### Animals and ethics statement

This study was carried out in accordance with the recommendations in the UK Animal (Scientific Procedures) Act 1986. The UK Home Office approved the study protocol (licence number 70/2716). The work was approved by the London-Hampstead NRES ethics committee REC reference 13/LO/0171.

All animals were housed in accordance with the UK Home Office guidelines: in dedicated rooms with controlled temperature, humidity, noise levels and lighting mimicking a natural sleep/wake cycle, and within individually ventilated cages with appropriate bedding and with *ad lib* access to food and water. All cages had nesting material and environmental enrichment. All animals were checked at least once a day by a competent person, any animals that raised concerns were checked by the Named Animal Care and Welfare Officer and if necessary the Named Veterinary Surgeon. All animals were euthanized using a rising concentration of CO_2_ with minimal animal suffering. No live animals were used for this project and organs were harvested *ex vivo* from mice. In order to minimise the number of animals, more than one organ was harvested from each animal according to the needs of other researchers in the institute. A total of 35 livers have been harvested *ex vivo* from 35 mice.

### Harvest of organs

Organs were obtained from CD-1 mice, aged 3 to 4 months. Livers were harvested as described in rats [26]. Briefly, after the sacrifice of the mouse using increasing CO2 inhalation, a midline incision was performed to expose the abdominal cavity. The portal vein (PV) was cannulated with a 24G cannula (BD, UK), secured with a 3/0 silk suture (Ethicon, UK). The inferior vena cava (IVC) was ligated using a 3/0 silk suture placed proximally to the right renal vein while the superior one was sectioned and left opened to allow the reagents to flow out. The diaphragm, once detached from the insertions, was used as holding point. The whole procedure was carried out to avoid damages to the Glisson’s capsule.

### Decellularisation protocol

Mouse livers were decellularised in a similar manner as rat livers using the Detergent Enzymatic Treatment (DET) method with a few modifications [26]. The PV was connected to a peristaltic pump (Masterflex, UK) and perfused with demineralised water (dH2O) (18.2 mΩ/cm) for 36 hours at 4°C followed by 4% sodium deoxycholate (SDC) (Sigma-Aldrich, UK) for 4h at room temperature (RT). Finally, after a 30 minutes wash with PBS, the liver was perfused with 2,000 kU solution of deoxyribonuclease-I (DNase-I, Sigma-Aldrich, UK) in 1 M sodium chloride for 3h at RT. All the reagents were perfused at a rate of 3 ml/min. The obtained mouse liver scaffolds were sterilised by gamma-irradiation before seeding cells.

### Cells and culture conditions

H1, a well-established embryonic stem cell line was used as hESCs line (WiCell Stem cell bank, WA01) [28]. The hiPSCs line was a generous gift from Prof. L. Vallier (corrected A1ATD-hiPSCs [29]). Stem cells were cultured in mTeSR1 medium (Stem cell technologies, UK) on matrigel growth factor reduced (Corning, UK) coated dishes. Media was changed daily and cells were split every 3–5 days with a mixture of collagenase IV and dispase II (Thermofisher, UK) [13]. All cells were tested monthly for the lack of mycoplasma contamination using the MycoAlert Mycoplasma Detection Kit (Lonza, UK).

### Differentiation protocol

hESCs and hiPSCs were differentiated into hepatocyte-like cells as described with a few modifications [12]. Stem cells were cultured until 80–90% confluency. They were split 1/6 on matrigel-coated dishes and let rest for 1–2 days with daily change of mTeSR1. They were then incubated with priming media Roswell Park Memorial Institute (RPMI) (Thermofisher, UK) and 1x B27 (ThermoFisher, UK) with 100 ng/ml activin A (Peprotech,UK) and 50 ng/ml Wnt3a (R&D systems, UK) for 3 days followed by 2-days incubation in 100 ng/mL activin A alone. At this point, Definitive Endoderm-like Cells (DECs) were harvested with enzyme-free Cell Dissociation Buffer (ThermoFisher, UK), avoiding remaining stem cells colonies and seeded in specification medium SR/DMSO (KO/DMEM containing 20% KO Serum Replacement, 1 mM L-glutamine, 1% NEAA, 0.1 mM β-mercaptoethanol, and 1% dimethyl sulfoxide) with rock inhibitor Y-27632 (Sigma-Aldrich, UK) for 1 overnight. For 2D cultures, DECs were seeded on matrigel-coated dishes: 6x10^4^ per well of a u-Slide 8 wells (Ibidi, Uk) or 5.4x10^5^ per 35 mm dish. For 3D cultures, the decellularised mouse liver was dissected into individual lobes and 2.5x10^6^ DECs in 200 μl of media were injected into 3 points of one decellularised lobe with a 29G microfine syringe (BD, UK), this was repeated 3 times drawing up the cells leaking out of the lobe after each injection. The seeded lobe was kept in a 24 wells plate with 500 μl of media and its size varied from 0.25–0.5 cm^3^. DECs, differentiated in parallel, were fixed and analysed afterwards by immunofluorescence staining for sox17, an endoderm marker. The media was then changed every 2 days for a further 4 days. During the final maturation step of 9 days, cells were fed every 2 days up to day 13 of differentiation with L-15 medium supplemented with 8.3% FBS, 8.3% tryptose phosphate broth, 10 μM hydrocortisone 21-hemisuccinate, 1 μM insulin (Sigma-Aldrich, UK), 2 mM glutamine, 10 ng/mL hepatocyte growth factor HGF (Peprotech, UK) and 20 ng/mL oncostatin M (Peprotech, UK). All media after the endoderm stage were supplemented with the antimicrobial Primocin (Invivogen, FR).

### DNA quantification

DNA was isolated using a tissue DNA isolation kit (PureLink Genomic DNA MiniKit, Invitrogen, UK) following the manufacturer’s instructions. The samples were digested overnight using Proteinase K. DNA samples were purified using alcohol washes and measured spectrophotometrically (Nanodrop, Thermo Scientific, US). Optical densities at 260 nm and 280 nm were used to estimate the purity and yield of nucleic acids. 4 biological replica were analysed.

### Scanning electron microscopy (SEM)

SEM-images of the cross-section, top and bottom surfaces of the decellularised scaffold were taken to examine surface-topography of the material. Samples were fixed in 3% glutaraldehyde (Sigma-Aldrich,UK; G5882) in 0.1m phosphate buffer and kept at 4°C. Scaffolds were first washed in 0.1m phosphate buffer (pH 7.4), then post-fixed with 1% Osmium tetraoxide (OsO_4_) / 1.5% Potassium Ferrocyanide K_4_(Fe(CN)_6_) in 0.1m phosphate buffer, followed by dH_2_O wash. Specimens were then dehydrated in a graded ethanol-water series to 100% ethanol and critical point-dried using CO_2_. Next, samples were mounted onto aluminium stubs using sticky carbon taps, so that the surfaces of interest were presented to the beam. Samples were coated with a 2nm-thin layer of Au/Pd using a Gatan ion-beam coater and viewed using a Jeol 7401 FEG-SEM.

### Samples preparation for immunostaining

To characterize the decellularisation of the mouse liver, fresh and DET liver were fixed in 4% paraformaldehyde PFA (Sigma-Aldrich, UK), dehydrated in graded alcohol, paraffin embedded and sectioned at 5μm.

After the differentiation protocol, for the 3D cultures, the liver lobes were embedded in OCT compound (VWR, UK) on a bath of isopentane (Sigma-Aldrich, UK) cooled in liquid nitrogen and sectioned at 6 μm on a cryostat. For the 2D cultures, cells were grown either on matrigel-coated u-Slide 8 well (Ibidi, Uk) or matrigel-coated 35 mm dishes and then fixed.

### Immunostainings: Haematoxylin & eosin, collagen, elastin, and glycosaminoglycans

Paraffin-embedded tissue sections were stained with haematoxylin and eosin (H&E; Leica, Germany), Picrosirius Red (Merck Sigma Aldrich., Germany) for collagen, Elastin Van Gieson (Millers Elastin, TCS Biosciences, UK) for elastin, and Alcian Blue (AB; BDH Chemicals Ltd, Cellpath Ltd) stains for glycosaminoglycans (GAG). Appropriate positive controls were used to ensure histological stains were performed correctly (small intestine for PR and AB, lung for EVG). For all histological samples, three biological and technical replicates were assessed using standard H&E prior to any special stains to ensure homogeneity. All images shown are representative samples.

For H&E staining, cryosections of 3D cultures and 2D cultured cells on matrigel-coated dishes were fixed in 4% PFA in PBS 15 min, rinse with PBS followed by tap water. They were incubated with Hematoxylin QS (Vector Laboratories, UK) 1–2 min, rinsed with tap water 5 min, and incubated with Eosin Y solution (Sigma-Aldrich, UK) for 30 sec. They were dehydrated with 95% then 100% EtOH, dipped in Histoclear (ThermoFisher scientific, UK) and mounted in a non-aqueous mounting medium.

### Lipid storage staining (Oil Red O)

Cryosections and cells on matrigel-coated dishes were fixed in 4% PFA in PBS 15 min. Samples were then incubated with filtered Oil Red O (ORO) working solution for 15 min (3 parts ORO stock solution (300 mg of ORO powder (Sigma-Aldrich, UK) into 100 ml of 99–100% isopropanol) with 2 parts demineralized water (dH2O)). Afterwards, they were rinsed with tap water, counterstained with Hematoxylin QS for 1 min and finally rinsed with tap water till the solution ran clear. Samples were kept dry or mounted in non-aqueous mounting medium.

### Glycogen storage staining (Periodic Acid Schiff)

Periodic Acid Schiff (PAS) was performed according to the manufacturer instructions (395B-1KT, Sigma-Aldrich, UK). Cryosections and cells on matrigel-coated dishes were fixed in 4% PFA in PBS 15–20 min. They were rinsed 3 times with tap water and incubated with Periodic Acid solution for 5 min. They were then rinsed with dH_2_O, 3 times for 5 min followed by an incubation with Schiff Reagent for 15 min, and finally rinsed 3 times with tap water for 5 min. Samples were counterstained with Hematoxylin QS for 1 min, rinsed with tap water and mounted in permanent mounting medium.

### Immunofluorescence stainings

Cryosections and cells on matrigel-coated dishes were fixed in 4% paraformaldehyde (PFA) for 15 min alone, methanol for 8–10 min alone or 4% PFA followed by methanol depending on the primary antibody. They were quenched with 10 mM NH_4_Cl for 10 min, permeabilised in 0.25% Triton X100 for 15 min, and blocked in appropriate blocking buffer for 1h. After overnight incubation with the primary antibody, samples were incubated with the appropriate AlexaFluor-conjugated secondary antibodies (S1 Table), counterstained with DAPI, treated 1 min with 0.1% Sudan Black B in 70% ethanol and mounted using Vectashield or Prolong Gold.

### Microscopy

All immunofluorescence images were acquired using Leica TCS SPE3 and SP5 confocal microscopes using 20x and 40x objectives. Leica Application Suite Advanced Fluorescence software was used for basic analysis of the confocal images. The images of ORO, PAS or H&E staining and bright field images of cells morphology were captured with an EVOS XL Core microscope using a 20x objective for the 2D cultures and Zeiss Axioplan2 microscope using a 20x and 40x objectives for the 3D cultures. Fiji software (Image J 1.50d) was used for counting the number of positively stained cells [30]. For nuclear staining, the threshold was adjusted so that the most of positive nuclei were visible for all images, then the plugin Analyze Particles was used with or without a Watershed filter. For cytoplasmic staining, the positive cells were counted manually with the help of the Cell Counter plugin.

### Statistics

For DNA quantification, a two-tailed Mann-Whitney test was performed as the normality of the population could not be assumed.

For analysis of the hepatocytes markers, a minimum of 3 separate differentiation experiments were analysed. The proportion of positively stained cells was analysed for each marker for each image (n) comparing the 4 groups: hESCs 2D or 3D and hiPSCs 2D or 3D (S2 Table).

The populations were represented by a boxplot of the percentages of positive cells for each marker for each group. The bottom and top of the box are the first (Q1 or 25^th^ percentile) and third quartiles (Q3 or 75^th^ percentile), and the band inside the box is the median. The ends of the whiskers represent the minimum and maximum. Mild outliers (○) are individual points with a value greater than Q3 + 1.5 x InterQuartile Range (IQR = Q3-Q1) or less than Q1–1.5 x IQR. Extreme outliers (▲) have a value greater than Q3 + 3 x IQR or less than Q1–3 x IQR.

All data points were included in the statistical analysis including outliers.

A two-tailed non-parametric Kruskal-Wallis test was performed because the populations could not be assumed to be Gaussian, as demonstrated by the Shapiro-Wilk normality test. This was followed by the Dunn Bonferroni posthoc test which enabled pairwise comparisons of the 4 groups. To aid the interpretation of the results, median, minimum and maximum were reported as well as mean rank for each group (S2 Table). The Mann-Whitney and Kruskal-Wallis tests compare medians if it can be assumed that the groups have similar distributions and compare mean ranks if this assumption cannot be made. The equality of variances among groups was tested with a non-parametric Levene test, and it was found that the groups did not have similar distribution for Alphafetoprotein (AFP), Cytokeratin 18 (CK-18), Cytokeratin (CK-19), and ORO measurements.

For all tests, a *p* value lower than 0.05 was considered significant. Statistical difference level on the graph was indicated by * for *p*<0.05 and ** for *p*<0.01 according to the results of the statistical test. All tests were performed using the statistical software SPSS v24 (IBM, US).

## Results

### Characterisation of mouse liver scaffolds

**Fig 1:**
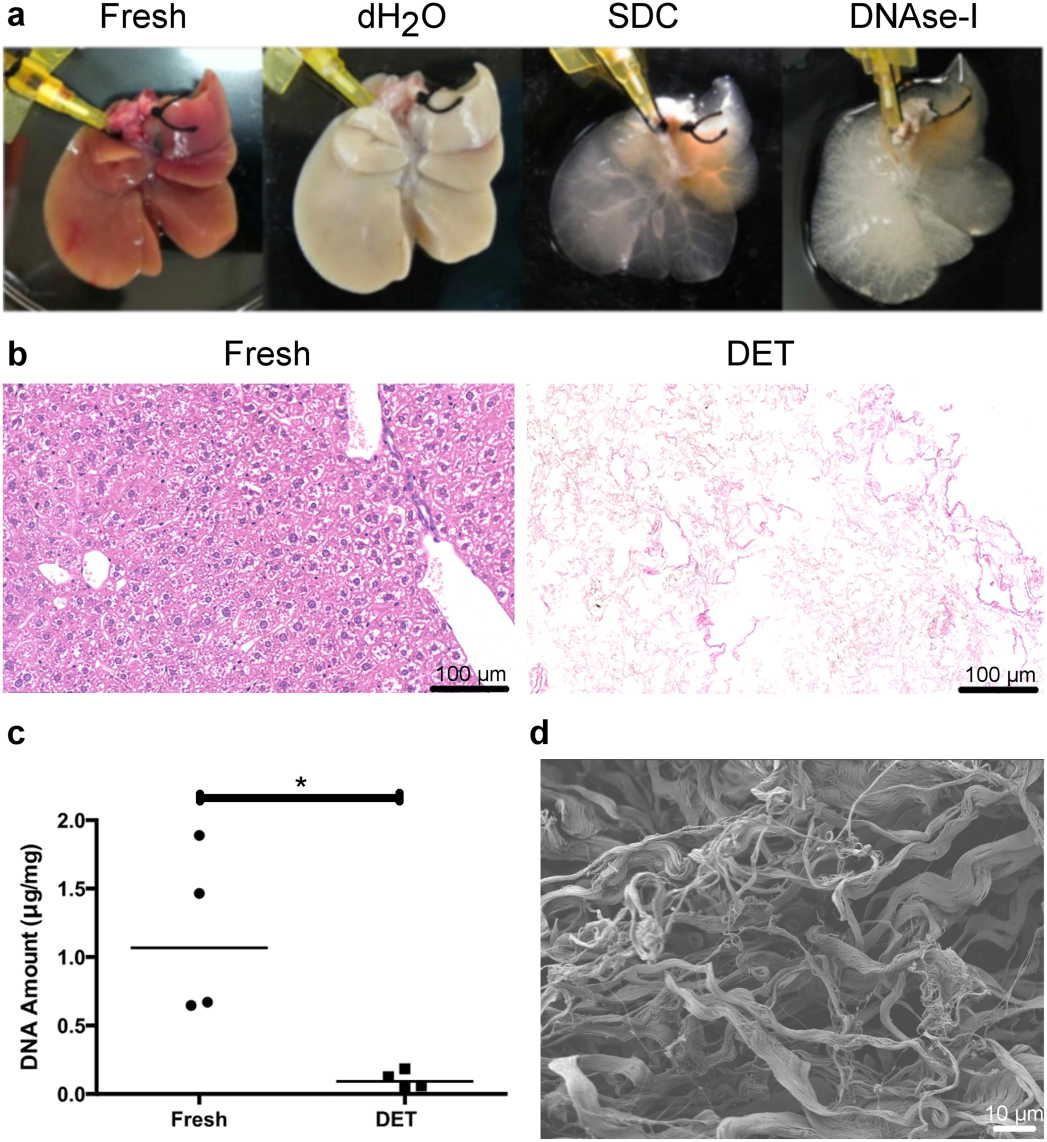
Mouse liver decellularisation. (a) Macroscopic appearance of a mouse liver scaffold during the decellularisation process. Following the perfusion with demineralized water (dH_2_O), the fresh red liver becomes blanched. After perfusion with sodium deoxycholate (SDC) followed by DNAseI solution, the liver becomes transparent. (b) H&E staining of the liver sections shows no cells in the scaffold after DET decellularisation. (c) DNA quantification shows a significant (*) reduction of DNA amount in the DET liver (median = 0.093 μg/mg) compared to fresh tissue (median = 1.068 μg/mg) (n_Fresh_ = 4, n_DET_ = 4; α = 0.05; p = 0.029; two-tailed Mann-Whitney test). (d) Scanning Electron Microscopy demonstrating the preservation of the three-dimensional network of connective tissue fibres.

**Fig 2:**
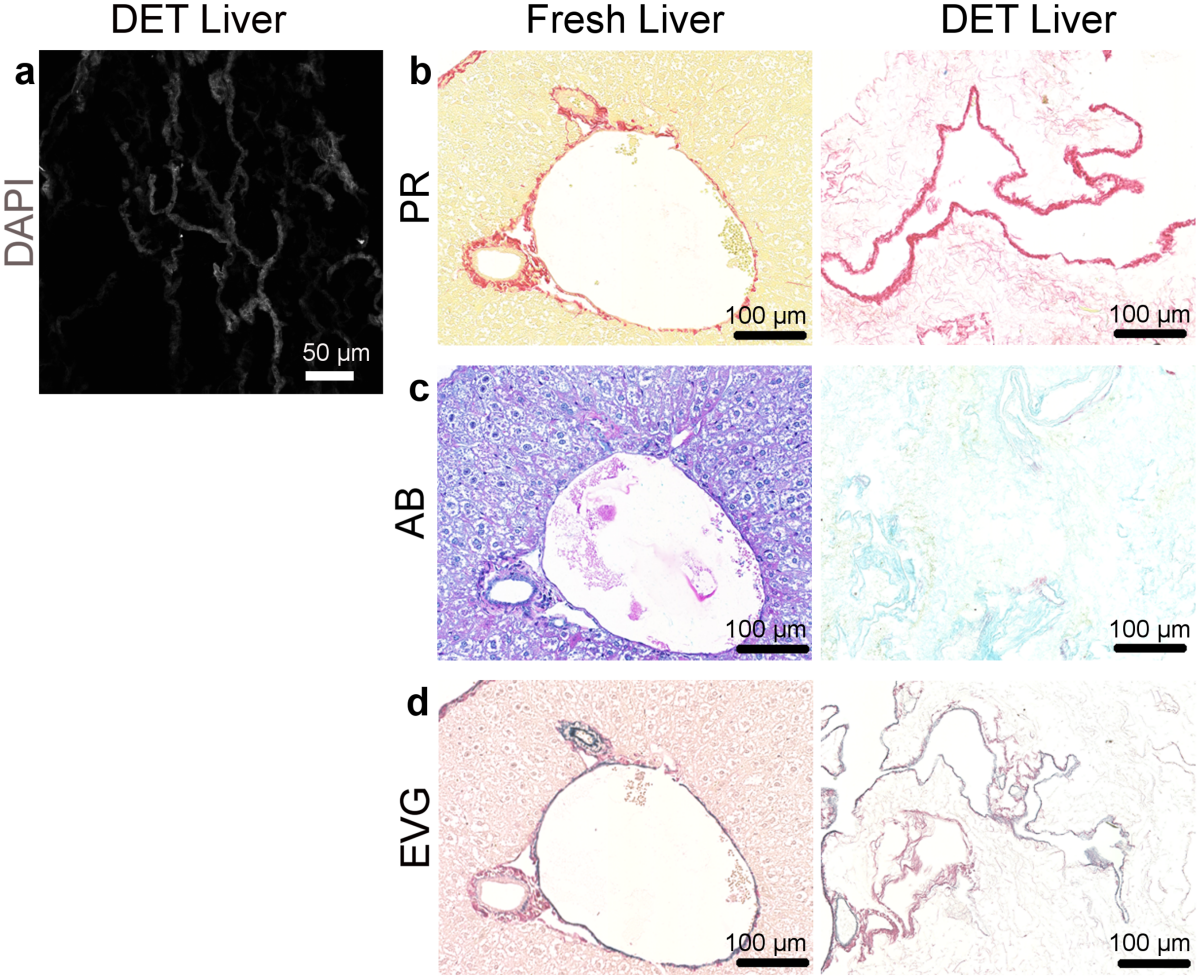
Characterisation of the mouse liver scaffold decellularised by DET method. (a) DAPI (grey) staining of sections of a DET mouse liver scaffold shows efficient decellularisation of the fibres with no nucleus present. (b) Picosirius red (PR) staining shows an abundance of collagen fibres in the scaffold. (c) Alcian Blue (AB) staining demonstrates presence of glycosaminoglycans (GAG). (d) Elastin van Gieson staining (EVG) confirms presence of elastin.

Decellularisation of the mouse livers was performed as described previously by detergent-enzymatic treatment (DET) [26]. By the end of the procedure, translucent livers were obtained (Fig 1a) with no detectable cells on H&E staining (Fig 1b) and minimal remaining DNA within the decellularised scaffold (Figs 1c and 2a). Scanning Electron Microscopy (SEM) showed the preservation of the three-dimensional network of connective tissue fibres (Fig 1d). The persistence of major ECM components such as collagen, elastin and glycosaminoglycans (GAGs) in the scaffold was confirmed by immunohistochemistry (Fig 2b–2d).

### Differentiation of hiPSCs and hESCs into hepatocyte-like cells within 3D or 2D cultures

hESCs and hiPSCs lines that had successfully been used to generate hepatocyte-like cells in 2D were utilised in this work [29, 31]. A previously established protocol was used for hepatocyte differentiation of stem cells [12, 32, 33]. hiPSCs and hESCs were first primed towards the definitive endoderm on matrigel-coated petri dishes. Definitive Endoderm-like cells (DECs) were then harvested using a non-enzyme cell dissociation buffer. The efficiency of differentiation towards endoderm cells was evaluated by immunofluorescence staining of sox17 positive cells. On 13 differentiation batches analysed, the highest percentage of sox17 positive cells per differentiation batch was 89.23±35% for hESC-derived endoderm and 91.42±5.77% for hiPSC-derived endoderm. On average 45.42±24.64% (hESCs) and 56.95±21.28% (hiPSCs) of cells were sox17 positive and were able to further differentiate into hepatocyte-like cells. As a low percentage of sox17 cells at endoderm stage predicts poor hepatocyte differentiation, the differentiation batches that were less than 15% sox17 positive were excluded from this study [13].

The DECs were then either seeded in 2D cultures or injected into several points of one lobe isolated from a mouse decellularised liver (3D cultures), which was then kept by itself in a petri dish in static conditions. The differentiation was then continued in parallel in 3D and 2D using hepatic specification stage media followed by the hepatic maturation stage-specific media.

**Fig 3:**
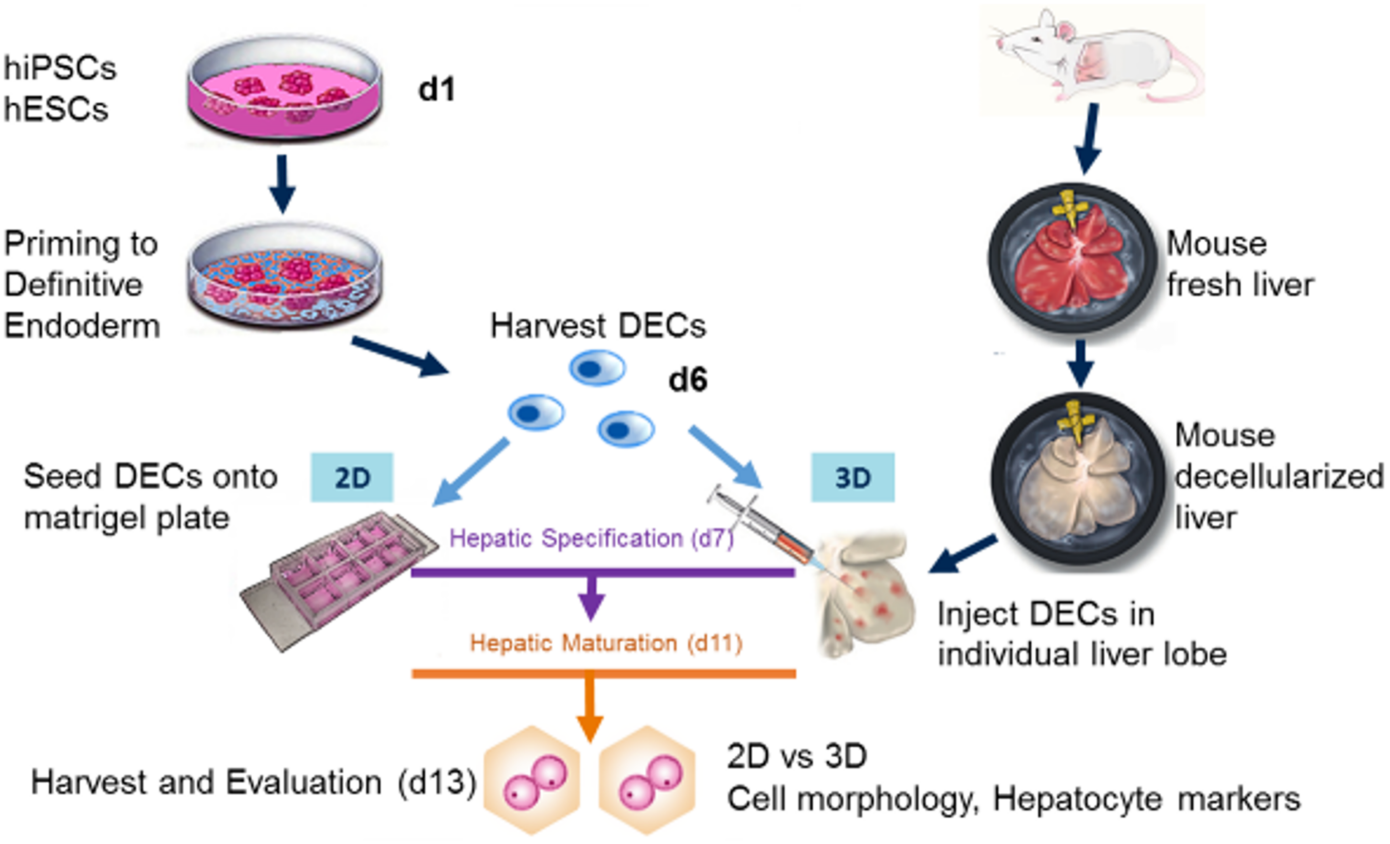
Differentiation of hiPSCs and hESCs in 2D or 3D cultures. hiPSCs and hESCs were differentiated first towards the definitive endoderm. Definitive Endoderm-like cells (DECs) were then harvested and either seeded on matrigel-coated petri dishes (2D cultures) or injected into multiple locations in individual decellularised liver lobes (3D cultures) on day 6. The differentiation protocol was continued in parallel in both systems with the hepatic specification stage from d7-d11 followed by the hepatic maturation stage from d11. The differentiation was continued up to day 13 when the samples were analysed. Part of the illustrations were published in Maghsoudlou et al [26].

For all experiments, the 2D and 3D cultures were compared on day 13 of differentiation (Fig 3, part of the illustrations were published in Maghsoudlou et al, figures 1 and 6 [26]).

### Repopulation of decellularised liver scaffolds by hiPSC- and hESC-derived cells

**Fig 4:**
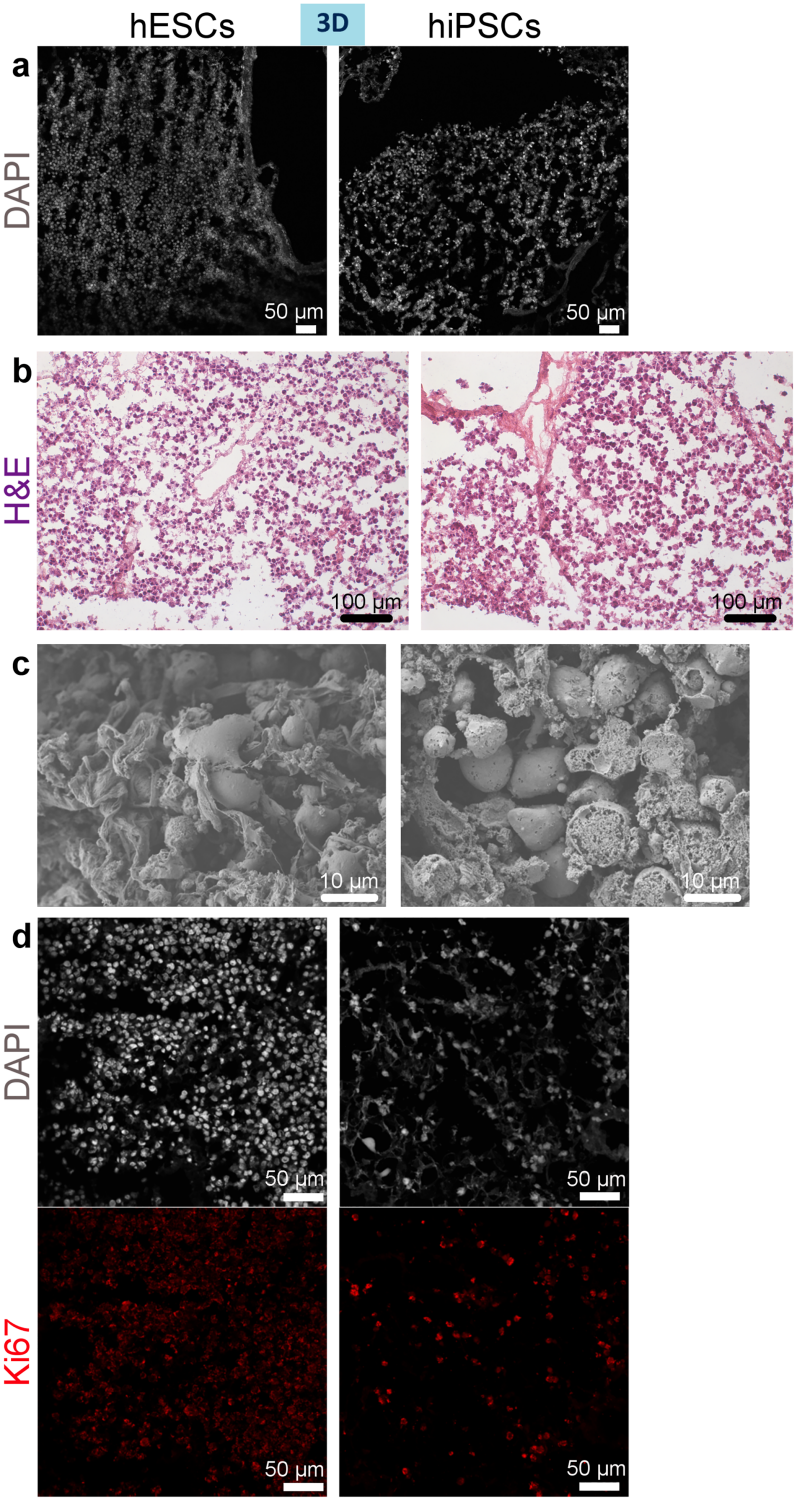
Repopulation of a mouse decellularised liver scaffold with hiPSC- and hESC-derived hepatocytes on day 13 of differentiation. (a-b) Nuclear staining by DAPI shows that cells have attached along the fibres of the scaffold (a) and that they repopulated the hepatic spaces as shown by H&E staining (b). (c) Scanning Electron Microscopy demonstrates engraftment of cells within the hepatic spaces of the scaffold. (d) Ki67 (proliferation marker) staining of sections of mouse liver scaffold repopulated with hESC- or hiPSC-derived hepatocytes. Nuclei counterstained with DAPI.

**Fig 5:**
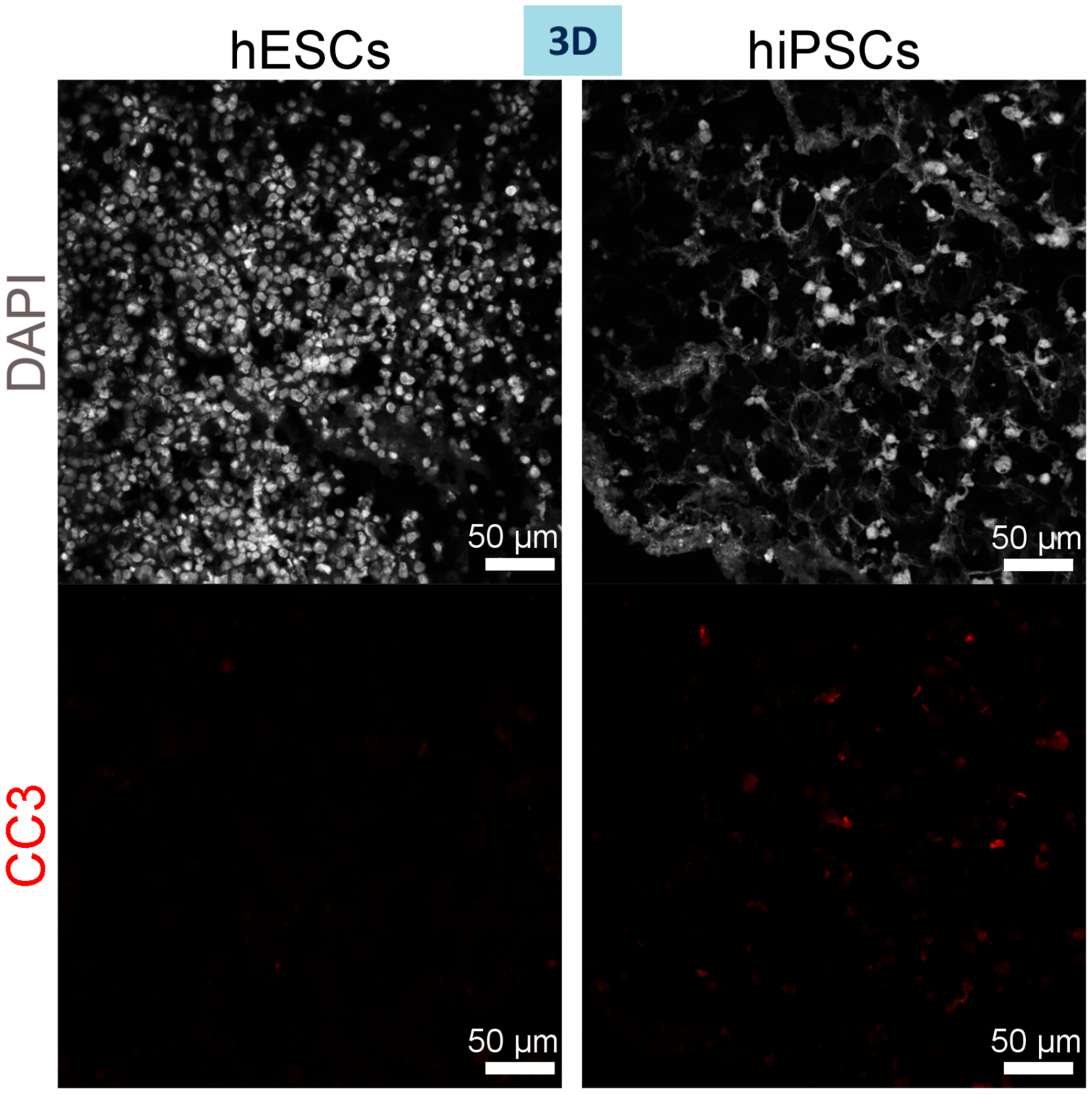
Minimal apoptosis amongst hiPSC- and hESC-derived hepatocytes cultured in 3D on day 13 of differentiation. Cleaved caspase 3 (red) staining demonstrates only occasional apoptotic cells. Nuclei stained with DAPI.

**Fig 6:**
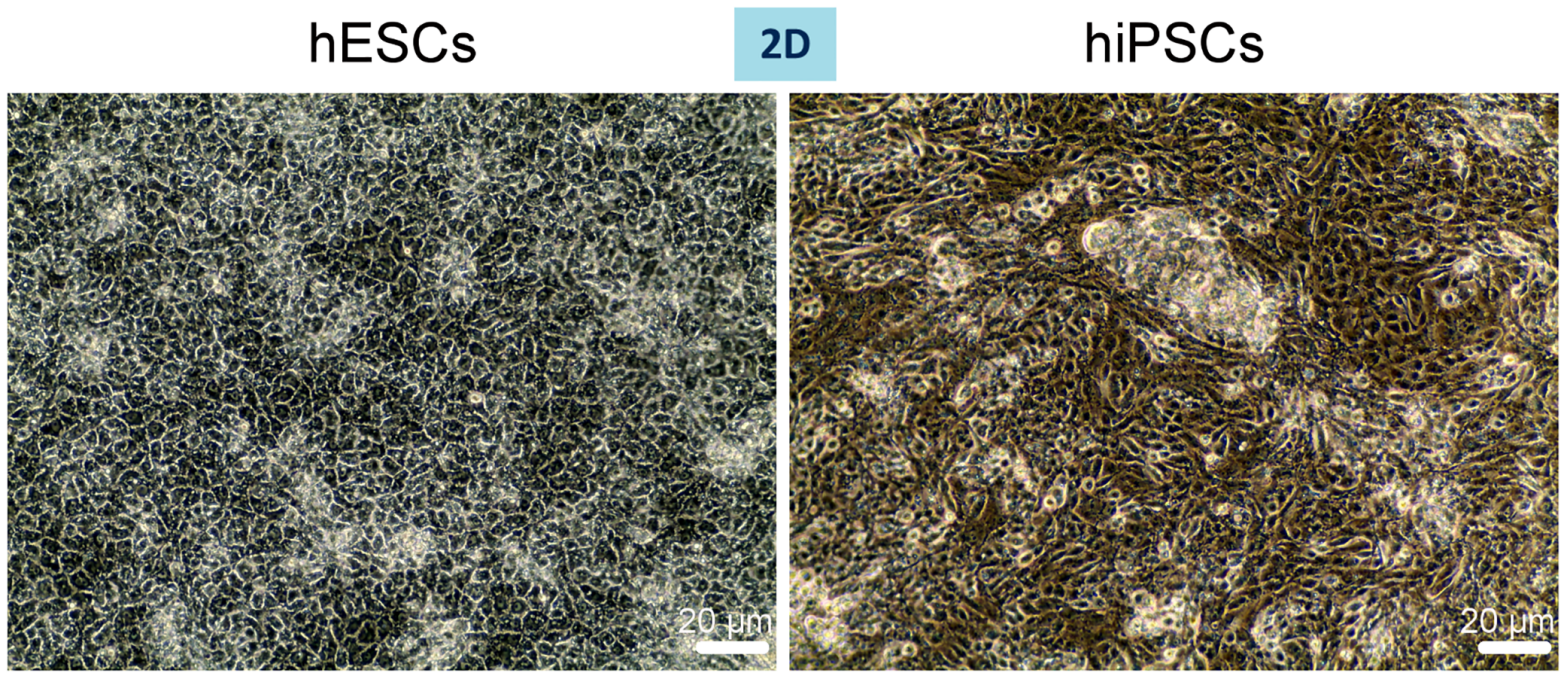
Morphology of hiPSC- or hESC-derived cells in 2D on day 13 of differentiation. Phase contrast microscopy shows cells starting to acquire a polygonal shape.

We first studied whether the cells were capable of engrafting within the scaffold. DAPI staining and H&E staining at a higher magnification of cryosections of the individually repopulated decellularised liver lobes demonstrated that hESC- or hiPSC-derived cells successfully repopulated the scaffolds and appear to be positioned along the scaffold fibres (Fig 4a and 4b). Scanning Electron Microscopy (SEM) of the 3D cultures revealed that cells were present within the hepatic spaces (Fig 4c). Ki67, a marker of proliferation shows substantial areas of cell proliferation (Fig 4d); while, cleaved caspase 3 (cc3), a marker of apoptosis, shows a minimal amount of apoptotic cells among the 3D cultured cells (Fig 5). 2D cultured cells start to acquire a polygonal shape on day 13 of differentiation (Fig 6).

### Earlier expression of mature hepatocytes markers in 3D cultured hiPSC- and hESC-derived hepatocytes

**Fig 7:**
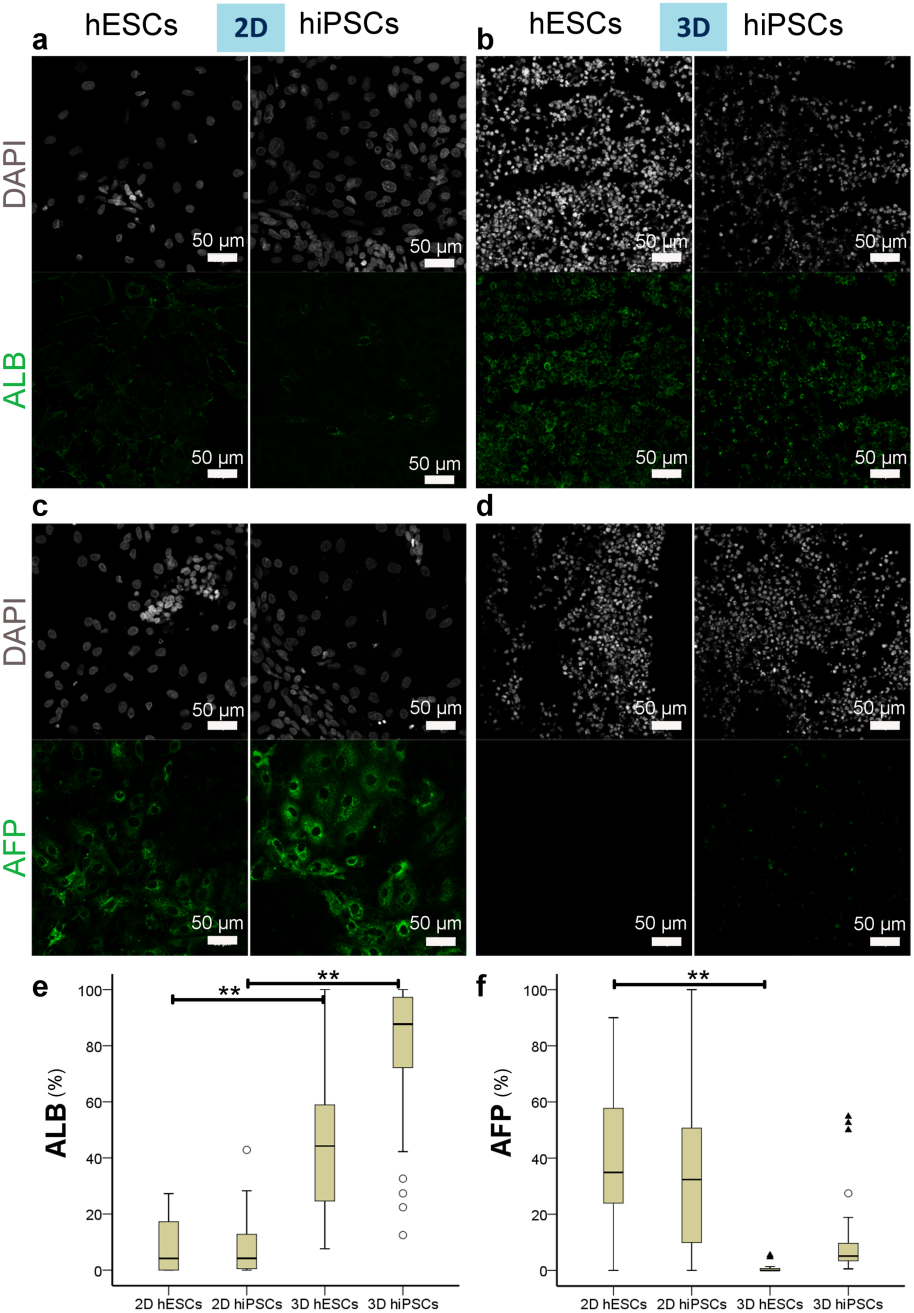
ALB and AFP expression by hiPSC- or hESC-derived hepatocytes on day 13 of differentiation. (a-b) Immunostaining of ALB shows more widespread expression in 3D (b) than in 2D (a). Nuclei stained with DAPI. (c-d) Immunostaining shows fewer cells expressing AFP in 3D (d) than in 2D (c). Nuclei stained with DAPI. (e-f) Boxplots of percentages of positive cells for ALB (e) and AFP (f) for each group. The bottom and top of the box are the first and third quartiles, and the band inside the box is the median. The ends of the whiskers represent the minimum and maximum. Individual data points represented by ○ and ▲ are mild and extreme outliers, respectively. Statistical difference level is indicated by ** for p<0.01 according to the results of the Dunn-Bonferroni posthoc tests performed with the Kruskal-Wallis test (α = 0.05).

Expression of characteristic hepatocyte markers was analysed on day 13 of differentiation in hiPSC- and hESC-derived cells. Positive cells for each marker were scored and reported as a percentage of the total number of cells identified by their positive DAPI staining of their nuclei for each image. Median expression of albumin (ALB) was 44.2% of the hESCs and 87.7% of the hiPSCs cultured in 3D, significantly different to only 4.15% for hESCs and 4.17% for hiPSCs cultured in 2D (p = 3.33E-16; α = 0.05; two-tailed Kruskal-Wallis test with Dunn-Bonferroni posthoc test). In particular, posthoc tests show that when comparing the expression of ALB between 3D and 2D culture conditions (p_2DhESCsvs3DhESCs_ = 2.44E-5, p_2DhiPSCsvs3DhiPSCs_ = 1.55E-11), the difference was found to be very significant (Fig 7a, 7b and 7e, S2 Table). Interestingly, there was no significant difference between hESC- and hiPSC-derived hepatocytes when cultured in 2D (p_2DhESCsvs2DhiPSCs_ = 1) but there was a significant difference between hESC- and hiPSC-derived hepatocytes when cultured in 3D (p_3DhESCsvs3DhiPSCs_ = 0.011).

On the contrary, median expression of alpha-fetoprotein (AFP) was 0% (hESCs) and 5.11% (hiPSCs) in 3D and was significantly different to 34.89% (hESCs) and 32.37% (hiPSCs) in 2D (p = 5.318 E-13; α = 0.05; two-tailed Kruskal-Wallis test with Dunn-Bonferroni posthoc test). According to the posthoc tests, the difference was very significant between hESC-derived hepatocytes cultured either in 2D or in 3D (p_2DhESCsvs3DhESCs_ = 1.704E-10) as well as between hESC- and hiPSC-derived hepatocytes grown in 3D (p_3DhESCsvs3DhiPSCs_ = 5.06E-6) (Fig 7c, 7d and 7f. S2 Table). Moreover, there was no significant difference between hESC- and hiPSC-derived hepatocytes when cultured in 2D (p_2DhESCsvs2DhiPSCs_ = 1), nor was there any significant difference between hiPSCs grown in 2D or in 3D (p_2DhiPSCsvs3DhiPSCs_ = 0.311). As the variances of the 4 groups for AFP are unequal (non-parametric Levene test, α = 0.05, p = 0.016), the Kruskal-Wallis test compares the mean ranks of the groups, rather than the medians. The mean ranks for hiPSCs grown in 2D or in 3D are quite similar; however, it is clear from their median expressions that they are biologically different.

Our results, therefore show that a higher percentage of 3D cultured stem cell-derived hepatocytes expressed ALB, a mature cytoplasmic hepatocyte marker, whereas expression of AFP, a fetal cytoplasmic hepatocyte marker, was much lower in 3D than in 2D cultured cells.

**Fig 8:**
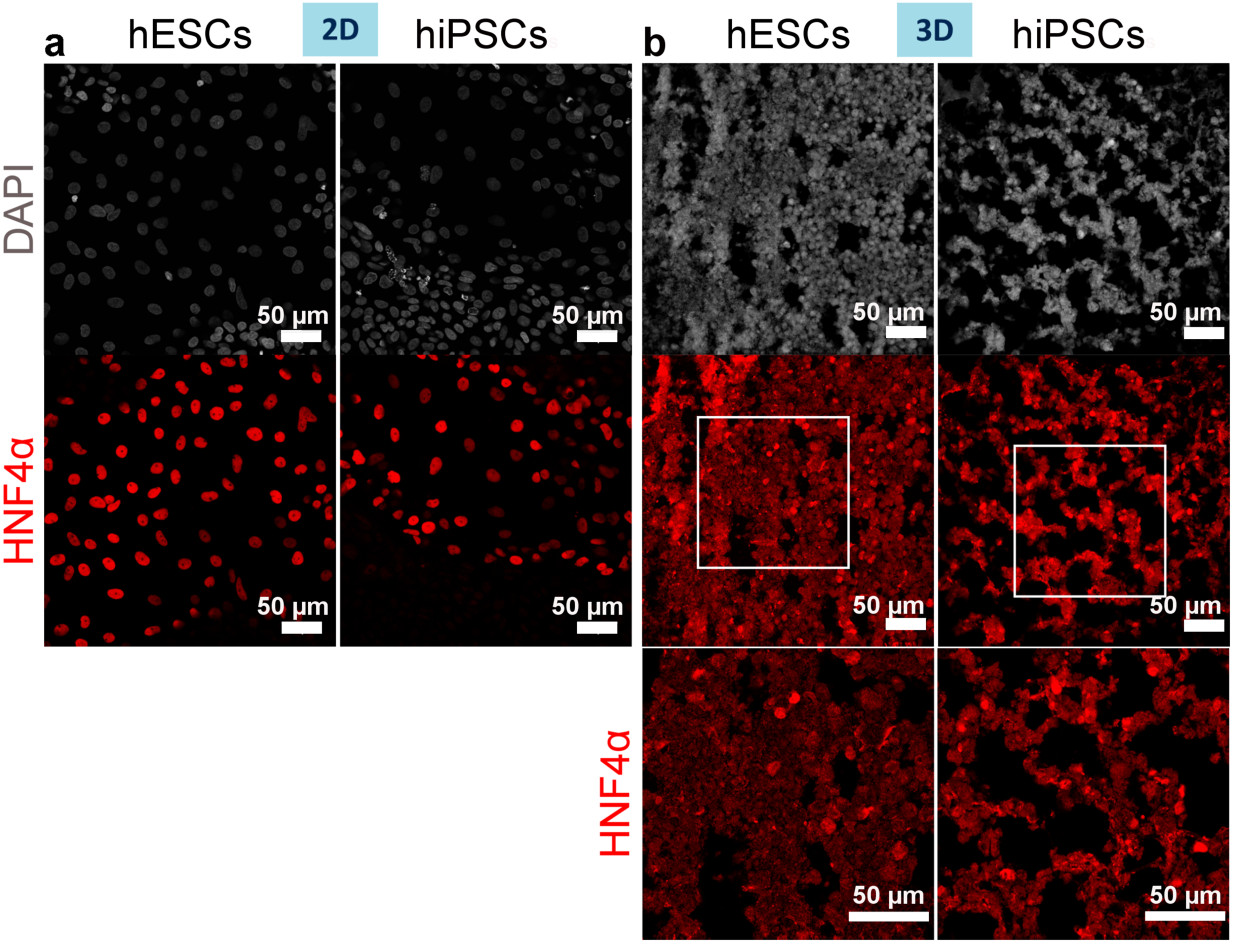
HNF4α expression by hiPSC- or hESC-derived hepatocytes on day 13 of differentiation. (a-b) Immunostaining of the hepatoblast and hepatocyte marker HNF4α shows its expression by 2D (a) and 3D (b) cultured cells. Nuclei stained with DAPI. The bottom high magnification panel corresponds to the delineated regions in lower magnification panel above.

HNF4α, a nuclear protein detected from early stages in hepatocyte differentiation and essential for specification of hepatocyte progenitors from pluripotent stem cells [34], was highly expressed in 2D cultured cells up to day 13, and was also present in cells grown on scaffolds (Fig 8).

We subsequently looked at intermediate filament proteins cytokeratin 18 (CK-18) and cytokeratin 19 (CK-19), which are both expressed at the hepatoblast stage of hepatocyte differentiation. In mature cells, CK-18 marks hepatocytes whereas CK-19 expression is specific to cholangiocytes [35, 36]. Median expression of CK-18 was 36.17% for the hESC-derived hepatocytes and 45.25% for the hiPSCs in 3D cultured cells and was significantly different to hESC-derived hepatocytes (1.24%) and hiPSCs (4.17%), respectively in 2D cultured cells (p = 1.948E-18; α = 0.05; two-tailed Kruskal-Wallis test with Dunn-Bonferroni posthoc test). Interestingly, posthoc tests show no significant difference between hESC- and hiPSC-derived hepatocytes when cultured in either 2D or 3D (p2DhESCsvs2DhiPSCs = 1, p3DhESCsvs3DhiPSCs = 1) within cell types when comparing 3D and 2D culture conditions (p2DhESCsvs3DhESCs = 3.37E-10, p2DhiPSCsvs3DhiPSCs = 3.08E-9). Even when CK-19 expression was evaluated, there were significant differences between the hESC-derived hepatocytes (1.63%) and hiPSCs (2.66%) in 3D and the same cells (both 0%) in 2D cultures (p = 0.003; α = 0.05; two-tailed Kruskal-Wallis test with Dunn-Bonferroni posthoc test, posthoc test:; p2DhiPSCsvs3DhiPSCs = 0.002). However, it is probable that there was no real biological difference between the 4 groups as CK-19 had low expression in all groups.

**Fig 9:**
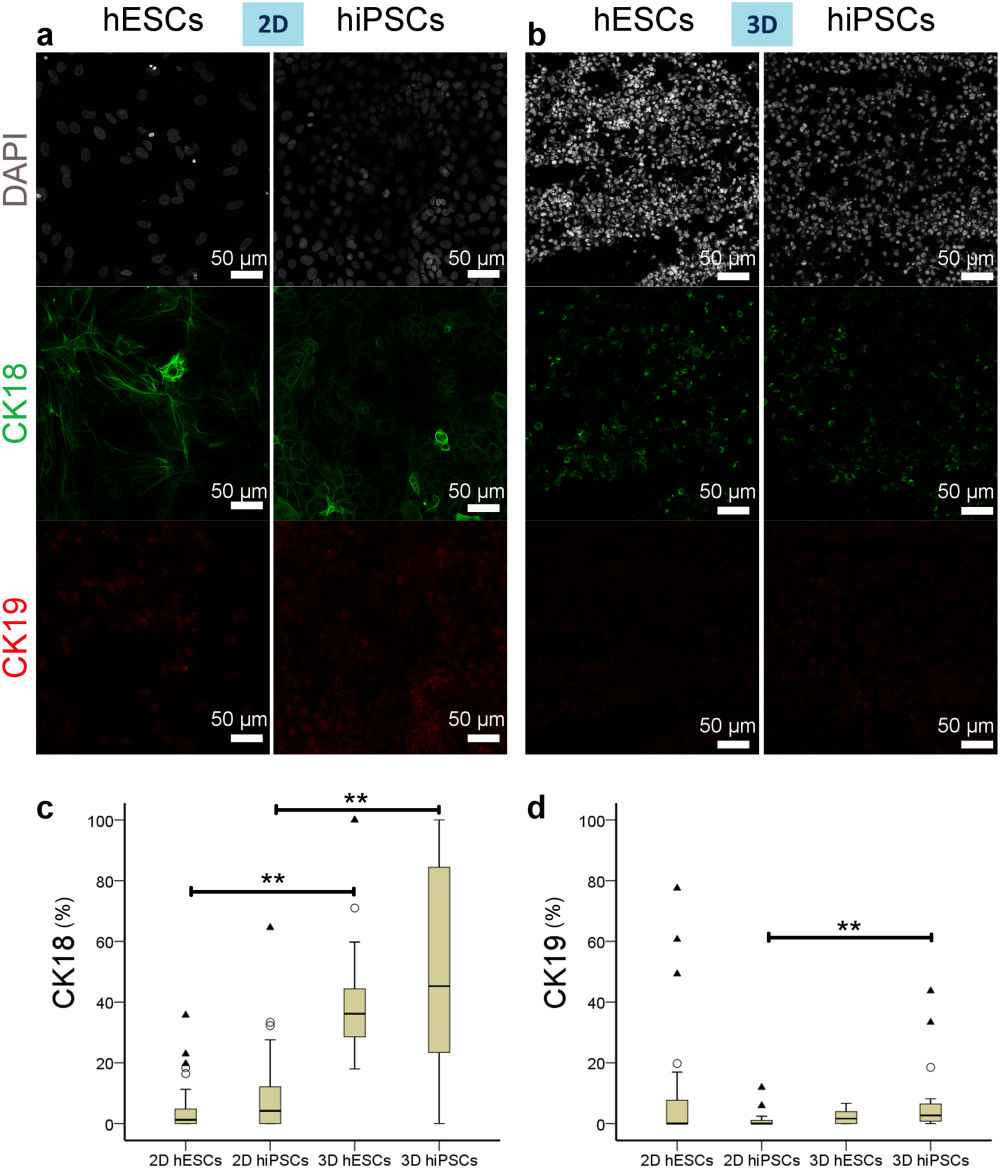
CK-18 and CK-19 expression by hiPSC- or hESC-derived hepatocytes in 2D and 3D on day 13 of differentiation. (a-b) Immunostaining of CK-18, typically expressed by hepatoblasts and hepatocytes and CK-19, expressed by hepatoblasts and cholangiocytes. Whilst CK-18 staining is seen in both 2D (a) and 3D (b) cultured cells, CK-19 staining is absent in 3D cultures. Nuclei stained with DAPI. (c-d) Boxplots of percentages of positive cells for CK-18 (**c**) and CK-19 (d) for each group. The bottom and top of the box are the first and third quartiles, and the band inside the box is the median. The ends of the whiskers represent the minimum and maximum. Individual data points represented by ○ and ▲ are mild and extreme outliers, respectively. Statistical difference level is indicated by ** for p<0.01 according to the results of the Dunn-Bonferroni posthoc tests performed with the Kruskal-Wallis test (α = 0.05).

**Fig 10:**
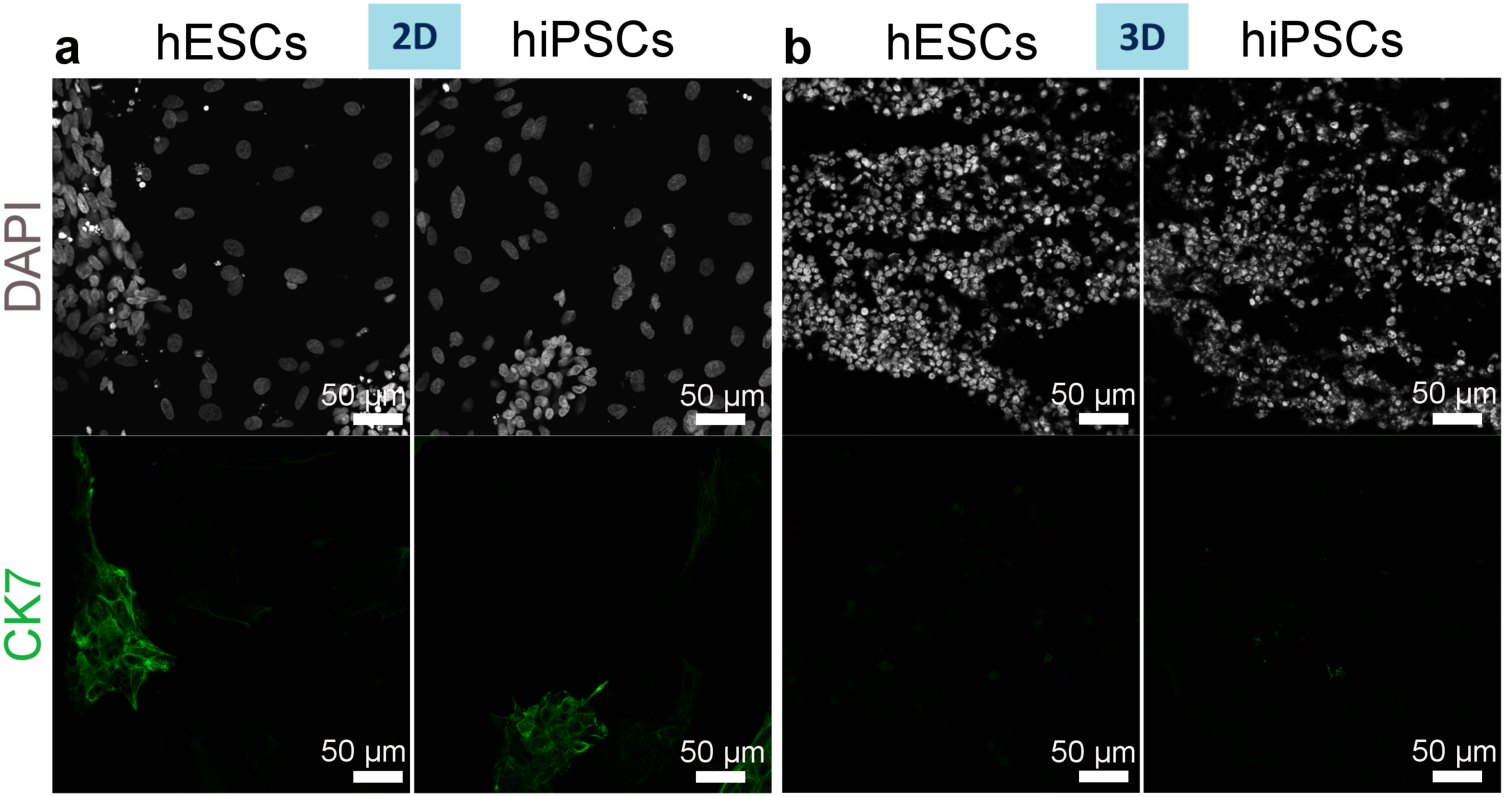
CK-7 expression by hiPSC- or hESC-derived hepatocytes on day 13 of differentiation. (a-b) Immunostaining of CK-7, typically expressed by ductal plate cells and cholangiocytes (CK7, green) demonstrates expression in 2D cultured (a) but not 3D cultured (b) cells. Nuclei stained with DAPI.

CK-18 was, therefore, highly expressed within the 3D cultures while expression of CK-19 and CK-7, another ductal plate and cholangiocyte marker [36] was very low. Within the 2D cultures, only occasional islands of cells expressing CK-18, CK-19 and CK-7 were seen (Figs 9 and 10, and S2 Table).

**Fig 11:**
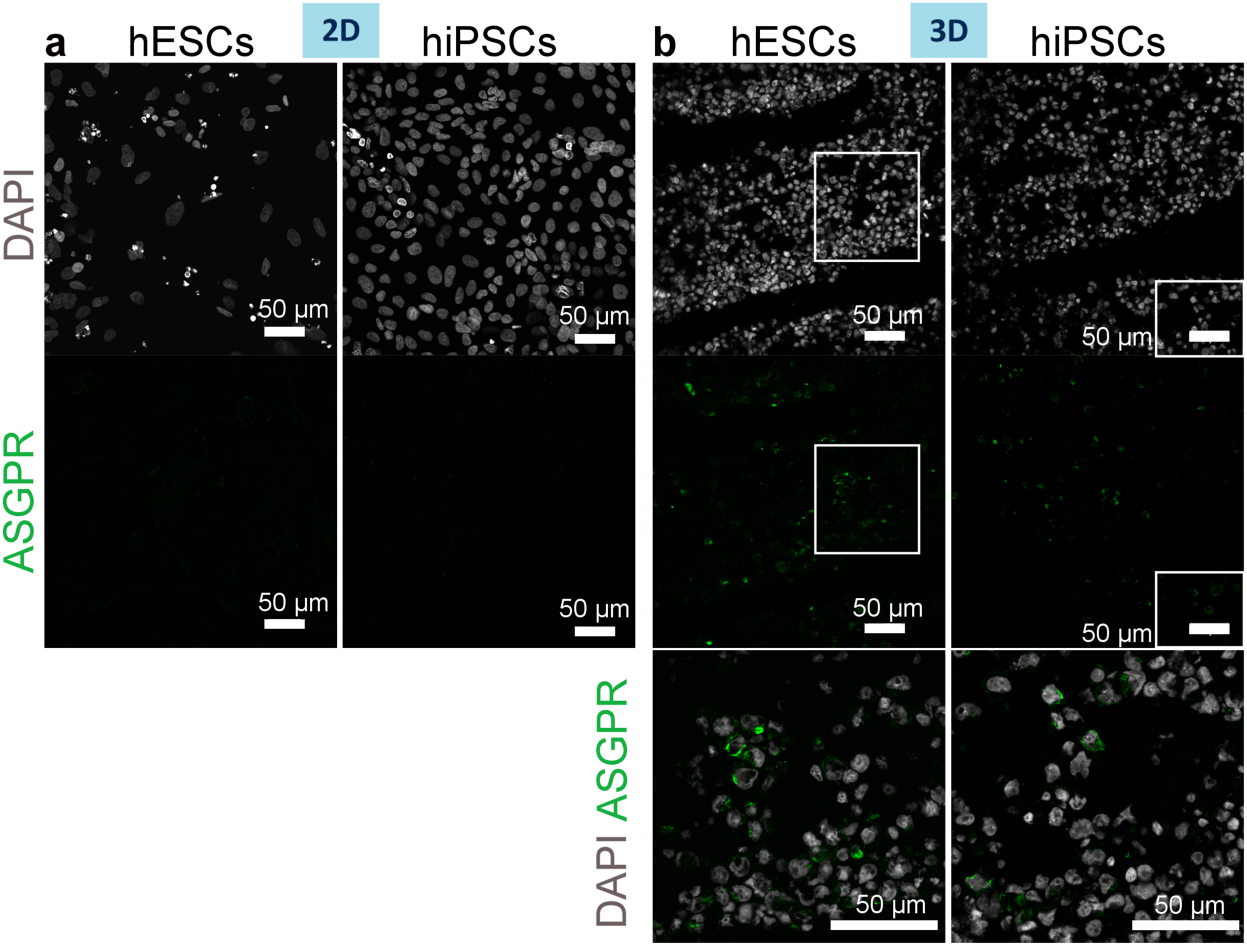
ASGPR expression by hiPSC- or hESC-derived hepatocytes in 2D or 3D on day 13 of differentiation. (a-b) Immunostaining of hepatocyte sinusoidal membrane protein ASGPR in 2D (a) and 3D (b) cultured cells. Nuclei stained with DAPI. The higher magnification images of merged channels in the bottom panel correspond to the delineated lower magnification images in the panel above. Images of 3D cultured cells demonstrate membrane localisation of ASGPR.

Interestingly, asialoglycoprotein receptor (ASGPR), which is normally localised at the sinusoidal membrane of the hepatocytes, was restricted to a part of the membrane in cells cultured in 3D, suggesting possible cell polarisation. No ASGPR expression was detected in cells cultured in 2D (Fig 11).

### A higher proportion of hiPSC- or hESC-derived hepatocytes are able to store lipids and synthesize glycogen in 3D than in 2D cultures

We then tested the ability of hESC- or hiPSC-derived cells to store lipids (Oil red O or ORO staining) and synthesize glycogen (Periodic Acid Schiff or PAS staining), these being primary hepatocyte functions.

**Fig 12:**
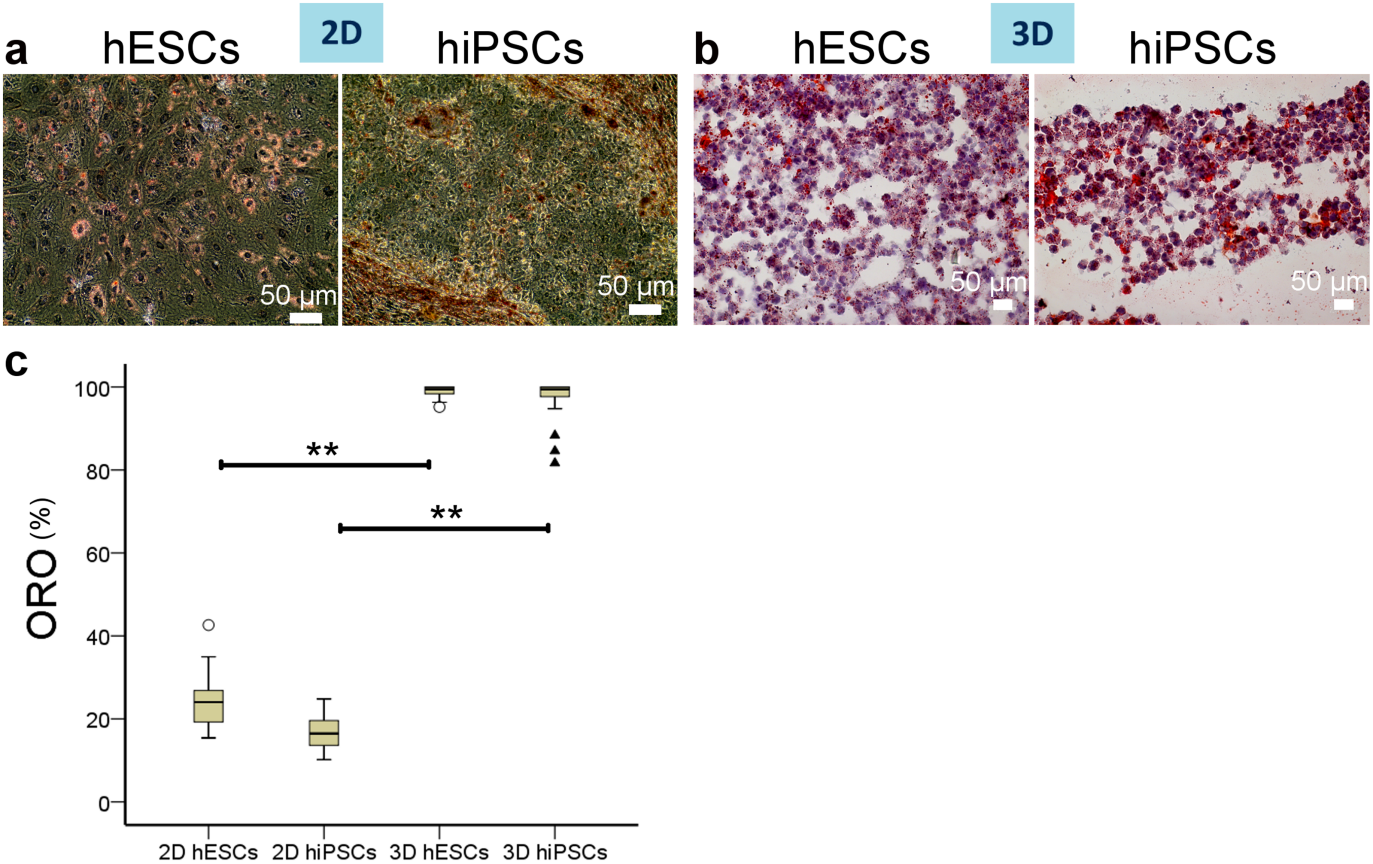
Lipid storage by hiPSC- or hESC-derived hepatocytes in 2D or in 3D on day 13 of differentiation. (a-b) Lipids detected by ORO (droplets, red-orange). Nuclei stained with hematoxylin (purple). Substantially higher percentage of cells are ORO positive in 3D (b) than in 2D (a) on day 13. (c) Boxplots of percentages of positive cells for ORO for each group. The bottom and top of the box are the first and third quartiles, and the band inside the box is the median. The ends of the whiskers represent the minimum and maximum. Individual data points represented by ○ and ▲ are mild and extreme outliers, respectively. Statistical difference level is indicated by ** for p<0.01 according to the results of the Dunn-Bonferroni posthoc tests performed with the Kruskal-Wallis test (α = 0.05).

Median expression of ORO was 99.5% for hESC-derived hepatocytes and 99.47% for hiPSC-derived hepatocytes in 3D at day 13; while only 24.03% (hESCs) and 16.47% (hiPSCs) of 2D differentiated cells were ORO positive (Fig 12).

The expression of ORO was significantly different between the 4 groups at day 13 of differentiation (p = 8.71E-15; α = 0.05; two-tailed Kruskal-Wallis test with Dunn-Bonferroni posthoc test). Posthoc tests show that there was no significant difference between hESC- and hiPSC-derived hepatocytes when cultured in 2D (p_2DhESCsvs2DhiPSCs_ = 1) nor in 3D (p_3DhESCsvs3DhiPSCs_ = 1). When comparing the expression of ORO between 3D and 2D culture conditions, the difference was found to be very significant (p_2DhESCsvs3DhESCs_ = 4.31E-5, p_2DhiPSCsvs3DhiPSCs_ = 6.17E-11) (Fig 12c and S2 Table).

**Fig 13:**
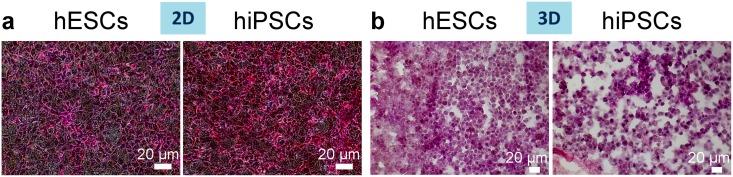
Glycogen synthesis by hiPSC- or hESC-derived hepatocytes on day 13 of differentiation. Periodic Acid Schiff staining (PAS, purple-magenta) is used to detect intracellular polysaccharides. A higher proportion of PAS positive cells is detected in 3D cultures (b) than in 2D cultures (a) at day 13 of differentiation. Nuclei were stained with hematoxylin (dark purple).

A higher proportion of hESC- and hiPSC-derived hepatocytes in 3D than in 2D showed a pink-purple staining due to the presence of polysaccharides indicative of their ability to synthesize glycogen (Fig 13).

Both assays indicate that a higher proportion of cells cultured in 3D are able to acquire hepatocyte-specific functions.

## Discussion

The high proliferation potential of stem cells and their ability to terminally differentiate into any specialised cell population makes them attractive for applications in regenerative medicine and disease modelling. However, one of the limitations to the use of stem cells as a source of hepatocytes is their inability to acquire a fully mature phenotype. Here we tested the utility of decellularised liver scaffolds for differentiation of both hESCs and hiPSCs.

We focused on scaffolds derived from adult mice livers to provide the adult ECM proteins in a 3D liver environment in an attempt to better mimic *in vivo* liver and improve hepatocyte maturation. We found that in this environment ALB was diffusely present in pluripotent stem cells after differentiation in 3D cultured cells while only a minimal fraction of the cells were positive when cultured in 2D. In parallel, the expression of the fetal hepatocyte marker AFP was negligible in 3D cultured cells while it was highly expressed in 2D cultures. These findings are consistent with the hypothesis that ECM scaffolds can promote cell differentiation. Beside phenotypic changes, we observed that 3D cultured cells appeared to be more functionally mature than the same cells maintained in 2D matrigel-coated dishes as shown by an increase in lipid storage and glycogen synthesis. Moreover, the 3D cultured cells demonstrated some polarised features such as localised ASGPR expression, which is also characteristic of and crucial to the function of mature hepatocytes.

Different batches of differentiating hESCs produced more consistent results than hiPSCs, and there were significant differences in staining for ALB and AFP detected between hESC- and hiPSC-derived hepatocytes mainly when grown in 3D, illustrating the intrinsic differences between stem cell lines.

Our finding of improved differentiation of stem cells on scaffolds is consistent with previous studies using different ECM components and 3D culture systems. Individual ECM proteins like laminins, have been shown to improve differentiation of pluripotent stem cells towards hepatocytes on 2D cultures [23]. Gieseck et al, in 2014 showed that a higher percentage of hiPSC-derived hepatocytes grown in 3D clumps are ALB, ORO and PAS positive than those cultured in 2D, and a lower percentage express AFP, which is consistent with our findings [20]. However, the authors used a longer protocol than the one utilised in this study with the phenotype of the hepatocytes studied at the end of the differentiation showing an improvement in maturation between 35 and 45 days of differentiation.

A number of improvements will be required to further optimise stem cell-derived hepatocyte maturation. Firstly, a high percentage of endoderm-like cells produced at the end of priming definitive endoderm stage is critical for successful hepatocytes differentiation [13], therefore improving the efficiency and consistency of endoderm production would improve the quality and reduce the variability between different differentiation batches. Co-culture of differentiating hepatocytes with endothelial, mesenchymal and other resident liver cells may further improve differentiation efficiency and hepatocyte maturity [37].

In our experiments, cells seeded onto scaffold did not survive until day 19, the end of the differentiation protocol. This finding is likely due to insufficient nutrient and oxygen supply provided to dividing cells in the scaffold in static conditions [38, 39]. Use of a bioreactor to bring a constant flow of oxygenated media would improve the survival of the cells grown within the scaffold which would allow further differentiation. Furthermore, the bioreactor grown cells could be subjected to the mechanical forces such as fluid shear stress which have been shown to promote stem cells endothelial differentiation and revascularisation of the scaffold [40–42].

In this work, we show that a decellularised liver scaffold is an effective tool for stem cell differentiation.

Production of high numbers of scaffold cultured mature hepatocytes will enable the introduction of drug screening assays not currently employed in the industry but that may significantly increase the repertoire of target diseases.

Several recent developments bring the idea of liver regeneration using stem cells closer to clinical application. The culture of pluripotent stem cells has recently been scaled up allowing the production of high cell numbers, necessary for application in clinical practice [43, 44].

Decellularised livers from various animal sources can be used as proteins preserved in the scaffold are unlikely to generate an immune reaction and therefore xenograft model may be accepted [45–47]. Thus the potential of liver scaffolds may in the future be utilised for terminal differentiation of stem cells prior to cell transplantation and even whole organ regeneration.

However, further studies are necessary to investigate the safety of hiPSC-derived hepatocytes, to improve the efficiency and consistency of differentiation, and to understand the long-term effect of the interaction between the repopulated cells, the ECM and the recipient.

## Supporting information

S1 Table List of Antibodies used, their dilution factor and blocking buffer used. (XLSX)

S2 Table Descriptive statistics for each hepatocyte marker for hiPSC- or hESC-derived hepatocytes in 2D and 3D cultures. (XLSX)
